# Silver(I) 2,2'-(1,2-Phenylenedisulfanediyl)diacetic Acid as a Molecular Building Block for a Silver(I)-Cadmium(II) Coordination Polymer

**DOI:** 10.3390/molecules20058020

**Published:** 2015-05-04

**Authors:** Ioana Georgeta Grosu, Christiane Berghof, Peter Lönnecke, Luminita Silaghi-Dumitrescu, Evamarie Hey-Hawkins

**Affiliations:** 1 Institut für Anorganische Chemie, Universität Leipzig, Johannisallee 29, D-04103 Leipzig, Germany; E-Mails: grosu.ioana@gmail.com (I.G.G.); Christiane.berghof@gmx.de (C.B.); loenneck@uni-leipzig.de (P.L.); 2 Department of Chemistry, Babeş-Bolyai University, Kogalniceanu 1, RO-400084 Cluj-Napoca, Romania; E-Mail: luminita.silaghi@googlemail.com

**Keywords:** 2,2'-(1,2-phenylenedisulfanediyl)diacetic acid, silver(I) complex, mixed Ag-Cd coordination polymer

## Abstract

Starting from heterotopic multidentate ligand 2,2'-(1,2-phenylenedisulfanediyl)diacetic acid, (*R*_S_,*R*_S_,*R*_S_,*R*_S_/*S*_S_,*S*_S_,*S*_S_,*S*_S_)-[Ag{1,2-C_6_H_4_(SCH_2_COOH)_2_-κ^2^*S,S'*}_2_]BF_4_ (**1**) was prepared and further used as a building block for the synthesis of heterobimetallic Ag-Cd coordination polymer [Ag_2_Cd_2_{1,2-(OOCCH_2_S)_2_C_6_H_4_}_3_(H_2_O)_3_ 5H_2_O]*_n_* (**2**). Both complexes were characterized by X-ray structure analysis and conventional spectroscopic techniques.

## 1. Introduction

Although the chemistry of coordination polymers has received much attention during the last decade, not only due to their interesting architectures [1] but also because of their potential applications in gas storage [2,3,4], nonlinear optics [5,6], or catalysis [7,8], and interesting magnetic [9,10] or luminescence [11] properties, the synthesis of such polymers with predictable geometries and structures is still a challenge [12,13]. Increasing attention has been paid in recent years to rational synthetic approaches for the assembly of target structures [14,15]. The key step of this approach is the design of molecular building blocks which can direct the formation of the desired architecture and functionality of the target compound.

Platinum(II) [16], manganese(II) [17,18,19], rhodium(II) [20], and copper(I) [21] complexes with bifunctional ligands were successfully used as “metalloligands” for rational construction of coordination polymers. Furthermore, heterobimetallic coordination polymers (e.g., Ln-Ba, Ln-Na, and Ln-Ca) that retain the luminescence properties of the mononuclear Ln complexes used as starting materials were obtained by a stepwise approach [22,23]. Nanoporous heterobimetallic Cd-Ag and heterotrimetallic Zn**-**Cd-Ag polymers were prepared from tris-chelate metalloligands (Cd, Zn) with 1,3-bis(4'-cyanophenyl)-1,3-propanedione as multifunctional chelate ligand and silver(I) salts [24]. Two one-dimensional heterotrimetallic Zn-Cd-Ag polymers with dicyanoargentate(I) bridges were obtained from a mixture of the metal salts and the nitronyl nitroxide radical ligand [25]; similarly, the mixed-ligand Ag-Cd heterometallic coordination polymer poly[bis(μ_3_-thiocyanato-κ^3^*N,S,S'*)(μ_2_-thiocyanato-κ^2^*N,S*)(4'-*p*-tolyl-2,2':6',2''-terpyridine-κ^3^*N,N',N''*)cadmium(II)silver(I)] was obtained from the corresponding metal salts and 4'-*p*-tolyl-2,2':6',2"-terpyridine [26].

Here, we report the synthesis of a mononuclear silver(I) complex from heterotopic multidentate ligand 2,2'-(1,2-phenylenedisulfanediyl)diacetic acid, which was further used as a molecular building block for the synthesis of a Ag-Cd heterobimetallic coordination polymer. 

## 2. Results and Discussion

### 2.1. Synthesis of **1** and **2**

**Scheme 1:**
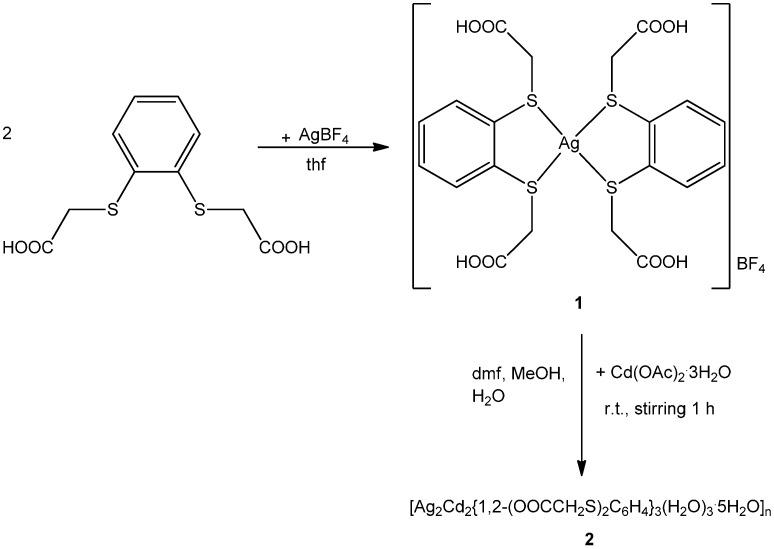
Synthesis of **1** and **2**.

With the goal of obtaining heterobimetallic coordination polymers through rational synthesis, the monomeric silver(I) complex of 2,2'-(1,2-phenylenedisulfanediyl)diacetic acid was prepared by heating the ligand with AgBF_4_ (2:1) in tetrahydrofuran (thf) for five minutes. The ligand 2,2'-(1,2-phenylenedisulfanediyl)diacetic acid coordinates via both sulfur atoms in a chelating manner, leaving the carboxyl groups uncoordinated. Thus, it should be possible to employ complex **1** as a building block to construct extended networks. Accordingly, **1** reacts with Cd(OAc)_2_ 3H_2_O (1:1) in dmf:H_2_O:MeOH (1:2:2.5) at room temperature for one hour to give heterobimetallic Ag-Cd coordination polymer **2** (Scheme 1).

Complex **1** was characterized by ^1^H, ^11^B{^1^H}, ^19^F{^1^H}, ^13^C{^1^H} NMR and IR spectroscopy and elemental analysis, and insoluble polymer **2** by IR spectroscopy and elemental analysis. Furthermore, single crystals suitable for X-ray crystallography could be obtained for both compounds.

### 2.2. Molecular Structures of **1** and **2**

**Figure 1:**
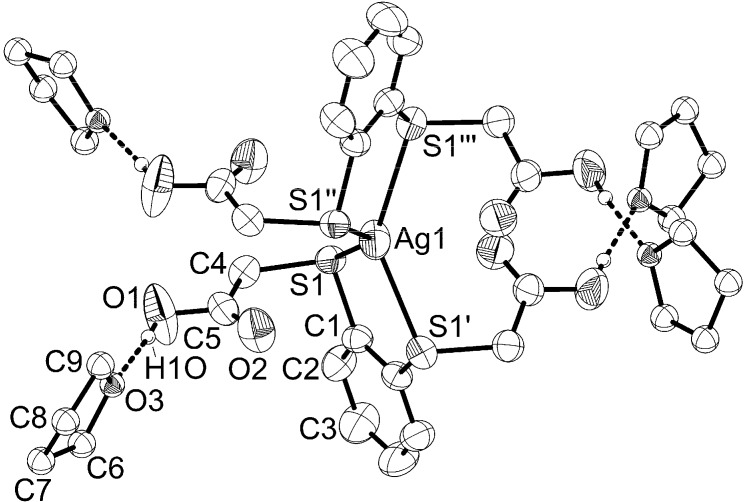
Molecular structure and atom labeling scheme for the silver complex cation in **1**. Each acetic acid proton (H1O) is hydrogen-bonded to a thf molecule. The BF_4_^−^ anion, CH protons, and noncoordinating thf molecules are omitted for clarity.

Suitable crystals of **1** for X-ray structure analysis were obtained from thf at −20 °C. Complex **1** crystallizes in the tetragonal space group $P\bar{4}$*n*2 with two molecules in the unit cell. In addition, eight thf molecules are also present in the unit cell. The structural motif of the [Ag{1,2-C_6_H_4_(SCH_2_COOH)_2_-κ^2^*S,S'*}_2_]^+^ cation in monomeric complex **1** is a distorted AgS_4_ tetrahedron with small S(1)–Ag(1)–S(1') bite angles (83.67(3)°) and large adjacent S(1)–Ag(1)–S(1'') (140.05(3)°) and S(1)–Ag(1)–S(1''') (110.10(3)°) bond angles. The metal center is located on a special position (three *C*_2_ axes) with site symmetry (222) [27] (Figure 1, Table 1).

**Table 1:** Selected bond lengths (Å) and angles (°) in **1**.

Ag(1)–S(1)	2.5518(8)	S(1)–Ag(1)–S(1')	83.67(3)
S(1)–C(1)	1.778(3)	S(1)–Ag(1)–S(1'')	140.05(3)
S(1)–C(4)	1.805(3)	S(1)–Ag(1)–S(1''')	110.10(3)
F(1)–B(1)	1.343(3)	F(1)–B(1)–F(1')	109.8(1)
O(1)–C(5)	1.307(4)		
O(1)–H(1o)	0.82(5)		
O(2)–C(5)	1.182(4)		
O(3)–C(9)	1.394(7)		
O(3)–C(6)	1.427(8)		

A distorted tetrahedral coordination geometry of silver ions was also observed in the related homoleptic discrete silver(I) complex cations [Ag{1,2-C_2_H_4_(SCH_2_Ph)_2_-κ^2^*S,S'*}_2_]^+^ (Ag–S 2*.*590(1) to 2*.*604(1) Å, S–Ag–S bite angles 85*.*17(5) and 86*.*58(5)°) [28] and [Ag{1,2-C_2_H_4_(SCH_2_CH_2_CH_2_COOH)_2_-κ^2^*S,S'*}_2_]^+^ (Ag–S 2*.*55(2) to 2*.*60(2) Å, S–Ag–S bite angle 86(1)°) [29]. The Ag(1)–S(1) bond length in **1** (2.5518(8) Å) is in the range of reported values [21,22,30]. The five-membered AgS_2_C_2_ rings are almost planar (deviation of C(1) and C(1') from plane: 0.02 Å). The sulfur atoms are in a tetrahedral environment (three substituents and one lone pair of electrons) and are thus chiral. Due to the presence of three perpendicular *C*_2_ axes at the silver center, all four sulfur atoms have the same configuration (either all *R* or all *S*).

The four carboxyl groups are noncoordinating, but each forms hydrogen bonds with one thf molecule (O(3)···H(1O) 1.80(5) Å, O(3)···O(1) 2.608(5) Å, O(3)···H(1O)–O(1) 174(5)°) (Figure 1). The BF_4_^−^ anion is located on a fourfold inversion axis with ideal tetrahedral symmetry.

Colorless crystals of **2** were obtained at room temperature in three days from a solvent mixture of dmf, MeOH, and H_2_O. Polymer **2** crystallizes in the monoclinic space group *P*2_1_ with two formula units in the unit cell. Five water molecules are present in the asymmetric unit.

**Figure 2:**
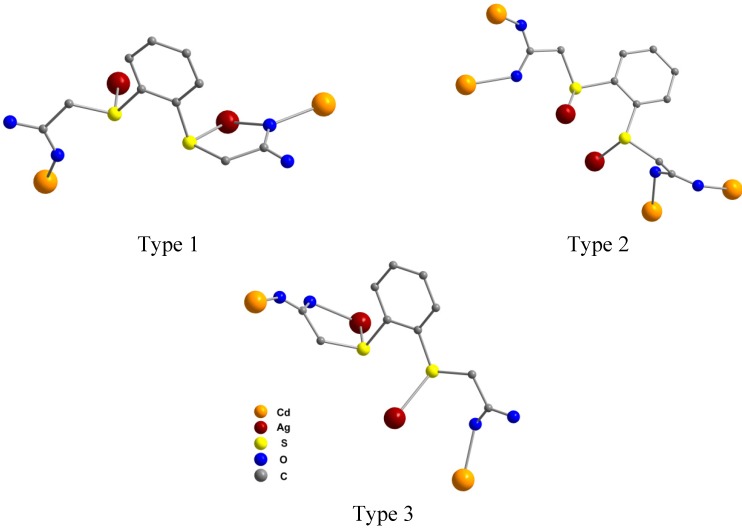
Observed coordination modes of C_6_H_4_(SCH_2_COO)_2_^2−^ in polymer **2**: tetradentate ligand (type 1), hexadentate (type 2), or pentadentate (in type 3).

Polymer **2** contains three types of C_6_H_4_(SCH_2_COO)_2_^2−^ anions (Figure 2) which differ from each other in their coordination mode with the metal ions (Cd^2+^ is exclusively coordinated by oxygen, whereas Ag^+^ is preferably coordinated by sulfur), as well as in the deviation of the SCH_2_COO groups from the plane of the benzene ring. The silver ions are no longer coordinated in a chelating fashion by four sulfur atoms of two neutral ligands but by three sulfur atoms from three different C_6_H_4_(SCH_2_COO)_2_^2−^ dianions and by one carboxylate oxygen atom.

In type 1, the C_6_H_4_(SCH_2_COO)_2_^2−^ anion acts as a tetradentate ligand, bridging two silver and two cadmium ions. Each sulfur atom coordinates to a silver ion. One of the silver ions is also coordinated in a chelating fashion by one carboxylate oxygen atom, which also coordinates to a cadmium ion. The second cadmium ion is coordinated by an oxygen atom from the second carboxylate group. One SCH_2_COO group is almost coplanar with the benzene ring, while the other is located out of the plane, and torsion angles C_aryl_–S–C_alkyl_–C are 169.16(1)° and –85.94(1)°, respectively. The type 2, a hexadentate C_6_H_4_(SCH_2_COO)_2_^2−^ anion bridges two silver and four cadmium ions, each oxygen atom being involved in coordination with a different cadmium ion, while each sulfur atom coordinates to a silver ion. Like in type 1, only one SCH_2_COO group is almost coplanar with the plane of the benzene ring, and the torsion angles C_aryl_–S–C_alkyl_–C are 165.04(1)° and –68.38(1)°. In the third type of coordination, type 3, the C_6_H_4_(SCH_2_COO)_2_^2−^ anion acts as a pentadentate ligand bridging two silver and two cadmium ions. Each sulfur atom coordinates to a silver ion. One of the silver ions is also coordinated in a chelating fashion by one carboxylate oxygen atom, and the second oxygen atom of this carboxylate group coordinates to a cadmium ion. The second cadmium ion is coordinated by an oxygen atom from the second carboxylate group. Again, one SCH_2_COO group is almost coplanar with the benzene ring, while the other is located out of the plane, and the torsion angles C_aryl_–S–C_alkyl_–C are –175.14(1)° and 81.22(1)°, respectively.

**Figure 3:**
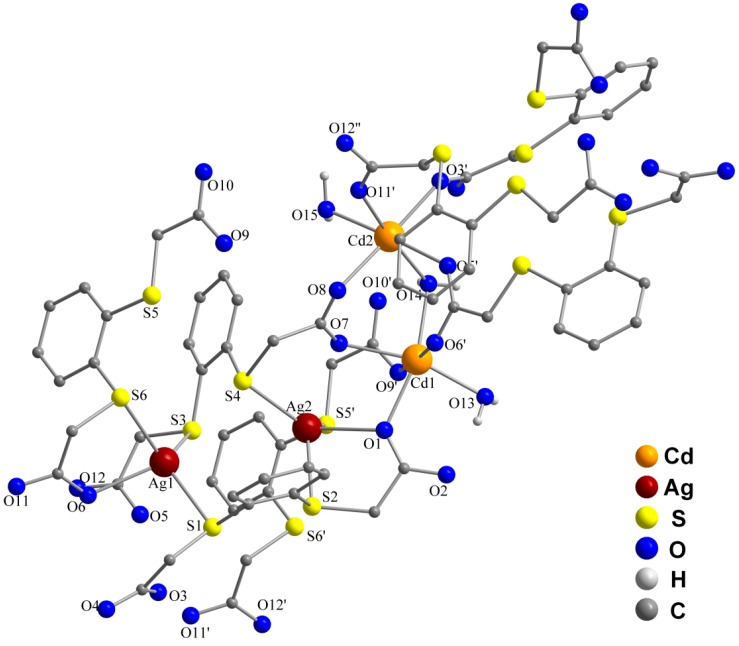
Coordination environments of the silver and cadmium ions and atom labeling in **2**. Hydrogen atoms other than O–H and the noncoordinating water molecules were omitted for clarity.

In **2**, two types of Cd^2+^ ions (Cd(1) and Cd(2)) and two types of Ag^+^ ions (Ag(1) and Ag(2)) are present. The Cd(1) ions are pentacoordinated in a square-pyramidal fashion by three carboxylate oxygen atoms from three different C_6_H_4_(SCH_2_COO)_2_^2−^ anions and by two oxygen atoms from two coordinating water molecules. The Cd(2) ions are hexacoordinated in a distorted octahedral fashion by four carboxylate oxygen atoms from four different C_6_H_4_(SCH_2_COO)_2_^2−^ anions and two oxygen atoms from two coordinating water molecules. The Cd(1) and Cd(2) ions are bridged by one oxygen atom (O(14)) from a water molecule which coordinates to both metal ions (Figure 3, Table 2). The Cd–O_carboxylate_ bond lengths range from 2.255(2) to 2.295(2) Å and are in agreement with those observed for similar compounds [31,32,33]. The Cd–O_water_ bond lengths vary from 2.247(2) Å (Cd(1)–O(13)) and 2.268(3) Å (Cd(2)–O(15)) for the terminal water molecules to 2.301(2) Å (Cd(1)–O(14)) and 2.344(2) Å (Cd(2)–O(14)) for the bridging water molecule.

**Table 2:** Selected bond lengths (Å) and angles (°) in **2**.

Cd(1)–O(1)	2.282(2)	O(1)–Cd(1)–O(7)	76.97(7)
Cd(1)–O(6')	2.255(2)	O(6')–Cd(1)–O(13)	90.66(8)
Cd(1)–O(7)	2.295(2)	O(7)–Cd(1)–O(13)	162.48(8)
Cd(1)–O(9')	2.275(2)	O(7)–Cd(1)–O(9)	79.70(7)
Cd(1)–O(13)	2.247(2)	O(9)–Cd(1)–O(13)	91.32(8)
Cd(1)–O(14)	2.301(2)	O(13)–Cd(1)–O(14)	107.07(8)
Cd(2)–O(3')	2.263(2)	O(3')–Cd(2)–O(8)	176.54(8)
Cd(2)–O(5')	2.319(2)	O(3')–Cd(2)–O(11')	92.15(8)
Cd(2)–O(8)	2.289(2)	O(3')–Cd(2)–O(15)	97.57(1)
Cd(2)–O(11')	2.260(2)	O(5')–Cd(2)–O(8)	91.68(8)
Cd(2)–O(14)	2.344(2)	O(8)–Cd(2)–O(15)	82.73(1)
Cd(2)–O(15)	2.268(3)	O(11')–Cd(2)–O(14)	174.21(8)
Ag(1)–O(12)	2.434(2)	O(12)–Ag(1)–S(1)	97.80(5)
Ag(1)–S(1)	2.534(8)	O(12)–Ag(1)–S(3)	124.95(5)
Ag(1)–S(3)	2.571(7)	S(1)–Ag(1)–S(3)	116.24(2)
Ag(1)–S(6)	2.592(8)	S(1)–Ag(1)–S(6)	126.86(2)
Ag(2)–O(1)	2.482(2)	O(1)–Ag(2)–S(4)	118.42(6)
Ag(2)–S(2)	2.604(8)	O(1)–Ag(2)–S(2)	73.15(5)
Ag(2)–S(4)	2.548(7)	S(2)–Ag(2)–S(5'))	120.37(2)
Ag(2)–S(5')	2.527(7)	S(4)–Ag(2)–S(2)	106.58(2)

Both silver ions Ag(1) and Ag(2) are tetracoordinated in a distorted trigonal-pyramidal fashion by two sulfur atoms from two different C_6_H_4_(SCH_2_COO)_2_^2−^ and by one sulfur atom and one carboxylate oxygen atom from a third dicarboxylate anion. The ions Ag(1) and Ag(2) are bridged by two sulfur atoms of the same ligand molecule (Ag(1)···Ag(2) 4.350(1) Å). The Ag–S bond lengths (2.527(7) Å to 2.604(8) Å) are in the same range as those of the starting material **1** (2.552(8) Å). The Ag–O bonds (2.434(2) Å and 2.482(2) Å) are shorter than those observed in *catena*-[(μ_2_-benzene-1,3-dicarboxylato)-bis(μ_2_-3,3',5,5'-tetramethyl-4,4'-bipyrazole)disilver(I)] (2.615(2) Å and 2.672(2) Å) [34].

**Figure 4:**
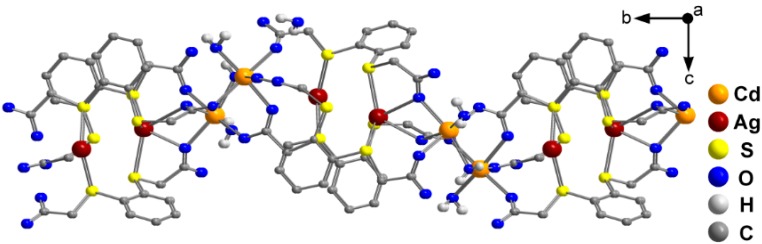
Two-dimensional layered structure of **2**, viewed along the *a* axis.

**Figure 5:**
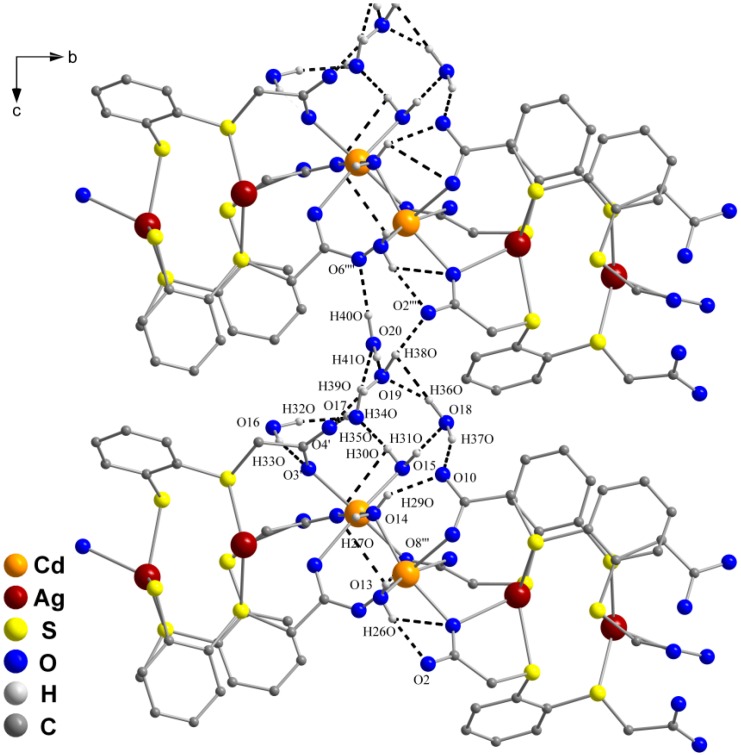

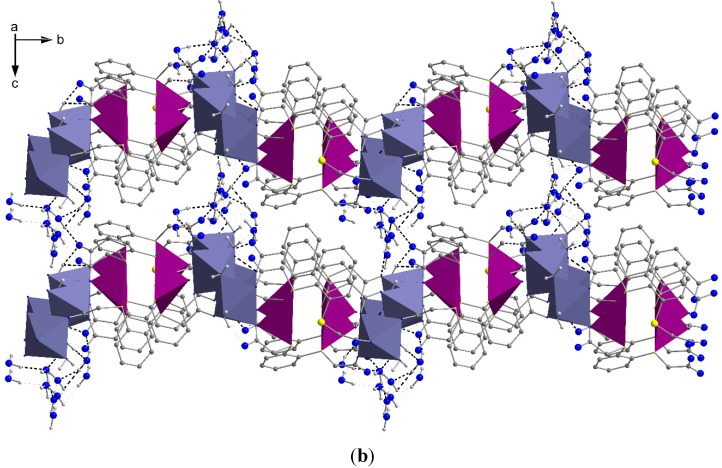
Three-dimensional hydrogen-bonded network of **2** (dashed lines) (**a**) with atom labeling and (**b**) with coordination polyhedra (blue-gray polyhedra denote the coordination environment of cadmium ions, and violet polyhedra the coordination environment of the silver ions).

The structure extends to a two-dimensional network parallel to the *C* face (Figure 4). The water molecules of solvation interconnect the two-dimensional sheets via hydrogen bonding, giving rise to a three-dimensional supramolecular network (Figure 5a,b). The five noncoordinating water molecules are involved in hydrogen bonding between themselves (H···O 1.69 Å to 1.93 Å and O···O 2.649(5) Å to 2.870(5) Å), with the water molecules coordinated to the cadmium ions (H(30O)···O(17) 1.93 Å, H(31O)···O(18) 1.69 Å, O(15)–H(30O)···O(17) 2.894(5) Å, O(15)–H(31O)···O(18) 2.649(5) Å), and also with carboxylate hydrogen atoms which belong to different sheets (H···O 1.81 Å to 2.07 Å and O···O 2.752(5) Å to 3.018(4) Å).

## 3. Experimental Section

### 3.1. General Procedures

All manipulations for the synthesis of compound **1** were carried out in an inert atmosphere of dry nitrogen and in the absence of light; the synthesis of **2** was performed in air. 2,2'-(1,2-Phenylenedisulfanediyl)diacetic acid [35,36] was prepared according to literature methods; AgBF_4_ and Cd(OAc)_2_ 3H_2_O were commercially available. The thf was dried over sodium/benzophenone, distilled under an atmosphere of dry argon, and stored over potassium mirror. CD_3_OD for NMR spectroscopy was kept in an inert atmosphere of dry argon over molecular sieves. The NMR spectra were recorded on a Bruker Avance DRX-400 spectrometer. Chemical shifts are quoted in ppm relative to tetramethylsilane. Elemental analysis was performed with a Vario EL-Heraeus microanalyzer. IR spectra were recorded with a Perkin-Elmer System 2000 in the range 4000–400 cm^−1^ in KBr pellets. 

### 3.2. Synthesis of (R_S_,R_S_,R_S_,R_S_/S_S_,S_S_,S_S_,S_S_)-[Ag{1,2-C_6_H_4_(SCH_2_COOH)_2_-κ^2^*S,S'*}_2_]BF_4_ (**1**)

A solution of AgBF_4_ (0.21 g, 1.08 mmol) in thf (20 mL) was added dropwise over a period of 5 min to a solution of 2,2'-(1,2-phenylenedisulfanediyl)diacetic acid (0.63 g, 2.44 mmol) in thf (15 mL). The reaction mixture was heated to reflux for 5 min. The solvent volume was reduced in vacuum to yield a white precipitate. Recrystallization from thf under a nitrogen atmosphere at −20 °C gave **1** (0.41 g, 38%) as air-stable colorless crystals. M.p. 73 °C. Elemental Anal. Calc. for C_20_H_20_AgBF_4_O_8_S_4_·4thf: C 43.25; H 5.24. Found: C 39.66; H 4.74%. The lower values indicate loss of thf (calculated for C_20_H_20_AgBF_4_O_8_S_4_·2thf: C 39.31; H 4.24) as was also observed by ^1^H-NMR spectroscopy. ^1^H-NMR (CD_3_OD): δ = 1.86 (m, 8H, CH_2_ in thf), 3.72 (m, 8H, CH_2_ in thf), 3.96 (s, 8H, SCH_2_), 7.44 (d, 4H, CH), 7.77 (d, 4H, CH). ^13^C{^1^H} NMR (CD_3_OD): δ = 25.1 (s, CH_2_ in thf), 38.8 (s, SCH_2_), 67.5 (s, CH_2_ in thf), 129.5 (s, CH), 133.0 (s, CH), 134.4 (s, SC), 170.7 (s, COOH). IR (KBr, cm^−1^): $\tilde{v}$ = 3440 (s, ν(O–H)), 2951 (s), 1705 (s, ν(C=O)), 1568 (w), 1431 (m), 1386 (m), 1318 (s), 1043 (s, ν(B-F)), 902 (m), 810 (m), 755 (s), 668 (m).

### 3.3. Synthesis of [Ag_2_Cd_2_{1,2-(OOCCH_2_S)_2_C_6_H_4_}_3_(H_2_O)_3_^.^5H_2_O]_n_ (**2**)

A solution of complex **1** (0.20 g, 0.28 mmol) and Cd(OAc)_2_ 3H_2_O (0.075 g, 0.26 mmol) in a mixture of dmf (4 mL), MeOH (10 mL), and H_2_O (8 mL) was stirred at room temperature for one hour. The reaction mixture was filtered and the resulting solution was kept unsealed in air. Colorless crystals of **2** (0.12 g, 44%) were formed in three days. The crystals were isolated by filtration and washed twice with H_2_O (10 mL). Elemental Anal. Calc. for C_30_H_30_Ag_2_Cd_2_O_15_S_6_^.^5H_2_O: C: 26.62; H: 2.98. Found: C: 25.68; H: 2.50%. IR (KBr, cm^−1^): $\tilde{v}$ = 3451 (s, ν(O–H)), 2930 (w, ν(CH)), 2364 (w), 2057 (w), 1620 (m), 1477 (w), 1384 (m, ν(C–O)), 1224 (w), 1042 (w), 900 (w), 813 (m), 754 (m), 691 (w), 638 (m), 561 (m), 523 (m). 

### 3.4. X-ray Structure Determination

Data for **1** were collected on a Siemens CCD diffractometer (SMART) [37] in ϕ-scan mode using Mo_Kα_ radiation (*λ* = 0.71073 Å). Data reduction was performed with SAINT [38], including the program SADABS [39] for empirical absorption correction. Data for **2** were collected on a CCD Gemini diffractometer (Agilent Technologies) in ω-scan mode using Mo_Kα_ radiation (*λ* = 0.71073 Å). Data reduction was performed with CrysAlis Pro including analytical numeric absorption correction using a multifaceted crystal model based on expressions derived by Clark and Reid [40]. All structures were solved by direct methods [41,42] and the refinement of all atoms was performed with SHELXL-97 [31]. With the exception of thf molecules, all hydrogen atoms for **1** were located in difference Fourier maps calculated at the final stage of structure refinement. For **2**, all H atoms except those of the water molecules H_2_O(13) and H_2_O(14) were calculated on idealized positions. Structure figures were generated with ORTEP [43] and DIAMOND-3 [44]. CCDC 877467 (**1**) and 877468 (**2**) contain the supplementary crystallographic data for this paper [45]. A summary of the data collection, structure solution, and refinement details for compounds **1** and **2** is given in Table 3.

**Table 3:** Data collection, structure solution, and refinement details for compounds **1** and **2**.

	**1**	**2**
Empirical formula	C_20_H_20_AgBF_4_O_8_S_4_ 4thf	C_30_H_30_Ag_2_Cd_2_O_15_S_6_ 5H_2_O
*M/*g mol^−1^	999.70	1353.52
*T/K*	213(2)	130(2)
Crystal system	tetragonal	monoclinic
Space group	$P\bar{4}$*n*2	*P*2_1_
*a/*Å	12.0071(6)	7.6790(1)
*b/*Å	12.0071(6)	24.2111(3)
*c/*Å	15.219(1)	11.7500(2)
*α*/°	90	90
*β*/°	90	102.175(1)
*γ*/°	90	90
*V*/Å^3^	2194.1(2)	2135.39(5)
*Z*	2	2
*D*_calcd_/Mg m^−3^	1.513	2.105
*μ*/mm^−1^	0.723	2.256
*F*(000)	1032	1332
Reflections collected	11088	48203
Independent reflections	2238 [R(int) = 0.0209]	12977 [R(int) = 0.0290]
Restraints/parameters	0/152	8/557
Goodness of fit on *F*^2^	1.061	0.985
Final *R* indices [*I* > 2 *σ*(*I*)]	*R*1 = 0.0310	*R*1 = 0.0227
	*wR*2 = 0.0756	*wR*2 = 0.0519
*R* indices (all data)	*R*1 = 0.0379	*R*1 = 0.0255
	*wR*2 = 0.086	*wR*2 = 0.0524
Largest diff. peak and hole/e Å^−3^	0.241 and −0.186	2.186 and −0.759
Absolute structure parameter	−0.01(4)	–0.02(1)

## 4. Conclusions

The reaction of 2,2'-(1,2-phenylenedisulfanediyl)diacetic acid with AgBF_4_ produced mononuclear silver(I) complex **1**, which was further used as a building block for the synthesis of two-dimensional heterobimetallic Ag-Cd coordination polymer **2**. In the discrete silver(I) complex cation in **1**, the four COOH groups are not coordinated to the metal center but are involved in hydrogen bonding with one thf molecule each. In **2**, the water molecules of solvation interconnect the two-dimensional sheets via hydrogen bonding, giving rise to a three-dimensional supramolecular network.

## References

[1] Cook T.R., Zheng Y.-R., Stang P.J. (2013). Metal-organic frameworks and self assembled supramolecular coordination complexes: Comparing and contrasting the design, synthesis and functionality of metal-organic materials. Chem. Rev..

[2] Eddaoudi M., Moler D.B., Li H., Chen B., Reineke T.M., O’Keeffe M., Yaghi O.M. (2001). Modular chemistry: Secondary building units as a basis for the design of highly porous and robust metal-organic carboxylate frameworks. Acc. Chem. Res..

[3] Robin A.Y., Fromm K.M. (2006). Coordination polymer networks with O- and N-donors: What they are, why and how they are made. Coord. Chem. Rev..

[4] Kitagawa S., Uemura K. (2005). Dynamic porous properties of coordination polymers inspired by hydrogen bonds. Chem. Soc. Rev..

[5] Noro S., Kitagawa S., Akutagawa T., Nakamura T. (2009). Coordination polymers constructed from transition metal ions and organic N-containing heterocyclic ligands: Crystal structures and microporous properties. Prog. Polym. Sci..

[6] Ho C.-H., Wong W.-Y. (2011). Facile tuning of photophysical traits and emerging applications in organic electronics and photonics. Coord. Chem. Rev..

[7] Bureekaev S., Shimomura S., Kitagawa S. (2008). Chemistry and application of flexible porous coordination polymers. Sci. Technol. Adv. Mater..

[8] Ma L., Abney C., Lin W. (2009). Enantioselective catalysis with homochiral metal-organic frameworks. Chem. Soc. Rev..

[9] Batten S.R., Murray K.S. (2003). Structure and magnetism of coordination polymers containing dicyanamide and tricyanomethanide. Coord. Chem. Rev..

[10] Kurmoo M. (2009). Magnetic metal–organic frameworks. Chem. Soc. Rev..

[11] Heine J., Müller-Buschbaum K. (2013). Engineering metal-based luminescence in coordination polymers and metal–organic frameworks. Chem. Soc. Rev..

[12] Cheetham A.K., Rao C.N.R., Feller R.K. (2006). Structural diversity and chemical trends in hybrid inorganic-organic framework materials. Chem. Commun..

[13] Shimizu G.K.H. (2005). Assembly of metal ions and ligands with adaptable coordinative tendencies as route to functional metal-organic solids. J. Solid State Chem..

[14] Kitagawa S., Kitaura R., Noro S.-I. (2004). Functional porous coordination polymers. Angew. Chem. Int. Ed..

[15] Ferey G. (2008). Hybrid porous solids: Past, present, future. Chem. Soc. Rev..

[16] Steto K.C., Kongshaung K.O., Jakobsen S., Tilset M., Lillerud K.P. (2008). Design, synthesis and characterization of a Pt-Gd metal-organic framework containing potentially catalytically active sites. Dalton Trans..

[17] Oh M., Carpenter G.B., Sweigart D.A. (2002). A coordination network containing metal-organometallic secondary building units based on π-bonded benzoquinone complexes. Chem. Commun..

[18] Oh M., Carpenter G.B., Sweigart D.A. (2003). A novel 3D brick-wall coordination network based on nodes with square-pyramidal connectivity. Angew. Chem. Int. Ed..

[19] Oh M., Carpenter G.B., Sweigart D.A. (2001). Metal-mediated self-assembly for π-bonded benzoquinone complexes into polymers with tunable properties. Angew. Chem. Int. Ed..

[20] Bickley J., Bonar-Law R., McGrath T., Singh N., Steiner A. (2004). Dirhodium (II) carboxylate complexes as building blocks *cis*-Chelating dicarboxylic acids designed to bridge the dinuclear core. New J. Chem..

[21] Lin W., Rieter W.J., Taylor K.L.M. (2009). Modular synthesis of functional nanoscale coordination polymers. Angew. Chem. Int. Ed..

[22] Chandler B.T., Cramb D.T., Shimizu G.K.H. (2006). Microporous metal-organic frameworks formed in a step-wise manner from luminescent building blocks. J. Am. Chem. Soc..

[23] Li M., Yuan L., Li H., Sun J. (2007). A 3D heterometallic metal–organic framework constructed from luminescent building blocks, exhibiting reversible dehydration and rehydration procedure. Inorg. Chem. Commun..

[24] Carlucci L., Ciani G., Maggini S., Proserpio D.M., Visconti M. (2010). Heterometallic modular metal-organic 3D frameworks assembled via new tris-β-diketonate metalloligands: Nanoporous materials for anion exchange and scaffolding of selected anionic guests. Chem. Eur. J..

[25] Wang S.-P., Li D.-J., Gao D.-Z., Chen J., Liu Z.-Q., Liao D.-Z., Jiang Z.-H., Yan S.-P. (2005). One-dimensional diamagnetic-metal nitronyl nitroxide radical complexes with dicyanoargentate(I) bridges: M(NIT4Py)_2_[Ag(CN)_2_]_2_ (M = Zn, Cd). Z. Anorg. Allg. Chem..

[26] Li Y.-Y., Wei Z.-H., Ng S.W. (2010). Poly[bis(*μ*_3_-thiocyanato-*κ*^3^*N*:*S*:*S*')(*μ*_2_-thiocyanato-*κ*^2^*N*:*S*)(4'-*p*-tolyl-2,2':6',2''-terpyridine-*κ*^3^*N*,*N',N''*)cadmium(II)silver(I)]. Acta Crystallogr. Sect. E: Struct. Rep. Online.

[27] Hahn T. (2005). International Tables for Crystallography.

[28] Li J.-R., Bu X.-H., Jiao J., Du W.-P., Xu X.-H., Zhang R.-H. (2005). Novel dithioether–silver(I) coordination architectures: Structural diversities by varying the spacers and terminal groups of ligands. Dalton Trans..

[29] Hittenhausen H., van der Meer H. (1978). Silver complex of 4,7-dithiadecane-1,10-dicarboxylic acid, C_20_H_35_AgO_8_S_4_. Cryst. Struct. Commun..

[30] Brunner H., Hollman A., Zabel M., Nuber B. (2000). The ligand [Cp_2_MoH_2_] in complexes with Ag-S bonds. J. Organomet. Chem..

[31] Gao S., Liu J.-W., Huo L.-H., Zhao H., Zhao J.-G. (2004). A one-dimensional chain Cd^II^ polymer: *Catena*-poly[[tris(1*H*-imidazole-κ*N**^3^*)cadmium(II)]-μ-benzene-1,4-dioxyacetato-κ^3^*O,O':O''*]. Acta Crystallogr. Sect. E: Struct. Rep. Online.

[32] Niu S.Y., Chi Y.X., Jin J., Yang G.D., Ye L. (2006). Synthesis and structure of the coordinatively unsaturated boron subphthalocyanine cation, [B(SubPc)]. Struct. Chem..

[33] Wang L., Yang M., Li G., Shi Z., Feng S. (2006). Synthesis and characterization of *f*-element iodate architectures with variable dDimensionality, α- and β-Am(IO_3_)_3_. Inorg. Chem..

[34] Hunger J., Krautscheid H., Sieler J. (2009). Hydrothermal synthesis and structure of coordination polymers by combination of bipyrazole and aromatic dicarboxylate ligands. Cryst. Growth Des..

[35] Rietmeijer F.J., Birker P.J.M.W.L., Gorter S., Reedijk J. (1982). Copper(I) and copper(II) chelates containing imidazole and thioether groups; Synthesis of the ligand 1,2 bis(benzimidazol-2'-ylmethylthio)-benzene (bbtp) and the X-ray crystal structure at −52 °C of [Cu(bbtp)(H_2_O)][ClO_4_]_2_·5EtOH. J. Chem. Soc. Dalton Trans..

[36] Cabiddu M.G., Cabiddu S., Cadoni E., de Montis S., Fattuoni C., Melis S., Sotgiu F. (1999). Metallation reactions. XXVI. α,α'-Dimetallation of 1,2-bis(methylthiobenzene). Tetrahedron.

[37] (1993). SMART, Area-Detector Software Package.

[38] (1999). SAINT, Area-Detector Integration Software.

[39] Sheldrick G.M. (1997). SADABS, Program for Scaling and Correction of Area-detector Data.

[40] Clark R.C., Reid J.S. (1995). The analytical calculation of absorption in multifaceted crystals. Acta Crystallogr. Sect. A: Found. Crystallogr..

[41] Sheldrick G.M. (2008). A short history of SHELX. Acta Crystallogr. Sect. A: Found. Crystallogr..

[42] Altomare A., Cascarano G., Giacovazzo C., Guagliardi A.J. (1993). Completion and refinement of crystal structures with SIR92. Appl. Crystallogr..

[43] Farrugia L.J. (1997). ORTEP-3 for Windows—A version of ORTEP-III with a graphical user interface (GUI). J. Appl. Crystallogr..

[44] Brandenburg K. DIAMOND 3—Crystal and Molecular Structure Visualization.

[45] Cambridge Crystallographic Data Centre. https://summary.ccdc.cam.ac.uk/structure-summary-form.

